# Influence of Acute and Chronic Exercise on Glucose Uptake

**DOI:** 10.1155/2016/2868652

**Published:** 2016-03-16

**Authors:** Martin Röhling, Christian Herder, Theodor Stemper, Karsten Müssig

**Affiliations:** ^1^Institute for Clinical Diabetology, German Diabetes Center, Leibniz Center for Diabetes Research, Heinrich Heine University Düsseldorf, 40225 Düsseldorf, Germany; ^2^German Center for Diabetes Research (DZD), Munich, 85764 Neuherberg, Germany; ^3^Department Fitness and Health, University Wuppertal, 42119 Wuppertal, Germany; ^4^Department of Endocrinology and Diabetology, Medical Faculty, Heinrich Heine University Düsseldorf, 40225 Düsseldorf, Germany

## Abstract

Insulin resistance plays a key role in the development of type 2 diabetes. It arises from a combination of genetic predisposition and environmental and lifestyle factors including lack of physical exercise and poor nutrition habits. The increased risk of type 2 diabetes is molecularly based on defects in insulin signaling, insulin secretion, and inflammation. The present review aims to give an overview on the molecular mechanisms underlying the uptake of glucose and related signaling pathways after acute and chronic exercise. Physical exercise, as crucial part in the prevention and treatment of diabetes, has marked acute and chronic effects on glucose disposal and related inflammatory signaling pathways. Exercise can stimulate molecular signaling pathways leading to glucose transport into the cell. Furthermore, physical exercise has the potential to modulate inflammatory processes by affecting specific inflammatory signaling pathways which can interfere with signaling pathways of the glucose uptake. The intensity of physical training appears to be the primary determinant of the degree of metabolic improvement modulating the molecular signaling pathways in a dose-response pattern, whereas training modality seems to have a secondary role.

## 1. Introduction

Insulin resistance plays a key role in the development of type 2 diabetes and is caused by genetic predisposition and environmental and lifestyle factors including physical inactivity and poor nutrition habits [1]. These risk factors also contribute to obesity, which is a major determinant of glucometabolic impairment and systemic subclinical inflammation [2]. Physical activity, as cornerstone in the prevention and treatment of diabetes, has marked acute and chronic effects on the regulation of glucose uptake and on inflammatory processes [3, 4]. The glucometabolic impairment in type 2 diabetes results from alterations of different signaling pathways modulating glucose uptake comprising insulin- and exercise-induced signaling pathways. However, during exercise, glucose uptake is normal or near normal [5], pointing to an insulin-independent activation of relevant signaling pathways mediating exercise-induced glucose uptake. An insulin-resistant state is also associated with changes in immunological and hormonal cross talk involving interleukin 6 (IL-6), tumor necrosis factor alpha (TNF-*α*), or adiponectin. These cytokines and adipokines are part of inflammatory processes and immune defense and can also affect molecular signaling pathways modulating glucose uptake. Behavioral interventions as well as unstructured physical activity have been shown to positively influence inflammatory processes, which was accompanied by improvements in glucose uptake [6, 7].

Physical exercise is distinguished primarily in resistance training and endurance training. Endurance training imposes a high-frequency (repetition), low-power output demand on muscular contraction, whereas resistance exercise imposes a low-frequency, high-resistance demand [8]. These two traditional modalities can also be performed as high-intensity training (HIT). This training form comprises alternating cycles of intensive and extensive phases involving endurance training, also known as high-intensity interval training (HIIT), and resistance training or the supramaximal exercise form of sprint interval training (SIT) [9, 10].

The overarching aim of this review is to summarize the mechanisms and molecular signaling pathways mediating glucose uptake as well as related changes in the release of immune mediators upon acute and chronic exercise exposure. Furthermore, we aim to assess the role of training intensity and training modality for the modulation of the aforementioned processes.

## 2. Search Strategy and Evaluation of Data

We searched PubMed/MEDLINE without language restriction from database inception until January 20, 2016, using the following search terms: “signaling OR pathway OR GLUT4 OR glucose OR inflammation OR inflammatory OR cytokine” AND “exercise OR training OR endurance exercise OR resistance exercise OR contraction”. Reference lists of review articles and all included articles identified by the search were also examined for other potentially eligible studies. The search was limited to human and animal studies. Duplicates were removed. Search results for relevant intervention studies are summarized in Table 1 and shown in detail in Tables 2, 3, 4, and 5.

The current literature does not provide a clear definition for acute or chronic effects of training [11]. The training effect is influenced by the time period between termination of the last bout of exercise and measurement as well as by training intensity [4]. Measurements of training effects within a time period of 0–72 h after exercise termination can show acute effects, even in a chronic training process, which makes it difficult to distinguish between acute and chronic training effects. In this review, we define the effect of chronic training as the sum of all training sessions, according to previous work [4].

## 3. Effect of Exercise on Molecular Signaling Cascades

### 3.1. Insulin Receptor Substrate 1 (IRS-1)/Phosphatidylinositol 3-Kinase (PI3-K) and Akt/Protein Kinase B (Akt/PKB) Pathways

**Figure 1:**
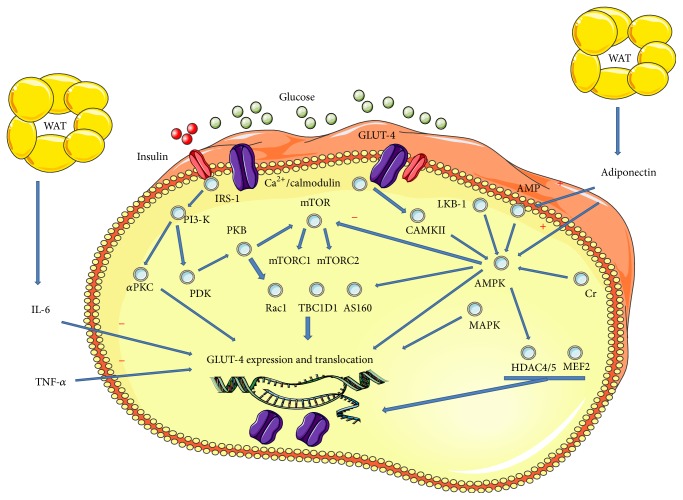
Interaction of important key players in exercise mediated glucose uptake of human muscle cells. A proposed model for the key players in glucose transport after physical exercise. *α*PKC, atypical PKC; AMP, adenosine monophosphate; AMPK, AMP-activated protein kinase; AS160, Akt substrate of 160 kDa; Ca, calcium; CaMKII, Ca^2+^/calmodulin-dependent protein kinase 2; Cr, creatine; GLUT-4, glucose transporter 4; HDAC4/5, histone deacetylase 4/5; IL-6, interleukin 6; IRS-1, insulin receptor substrate 1; LKB-1, liver kinase B1; MEF2, myocyte enhancer factor-2; MAPK, mitogen-activated protein kinases; mTOR, mammalian target of rapamycin (C1 complex 1 and C2 complex 2); PDK, phosphoinositide-dependent kinase; PI3-K, phosphoinositide 3-kinase; PKB, protein kinase B; Rac1, ras-related C3 botulinum toxin substrate 1; TBC1D1, TBC1 domain family member 1; TNF-*α*, tumor necrosis factor alpha; WAT, white adipose tissue.

In conditions of rest, insulin regulates glucose transport into the muscle due to activation of a protein signaling cascade. After binding of insulin to its receptor, the insulin receptor is autophosphorylated. Insulin receptor substrate 1 (IRS-1) binds to the phosphorylated tyrosine residues of the insulin receptor and is subsequently phosphorylated by the tyrosine kinase of the insulin receptor. Binding of IRS-1 to the p85 subunit of phosphatidylinositol 3-kinase (PI3-K) results in activation of a PI3-K-dependent pathway comprising phosphoinositide-dependent kinase (PDK) and atypical protein kinase C (*α*PKC) [110]. Key downstream molecules modulating translocation of glucose transporter type 4 (GLUT4) to the plasma membrane comprise, besides Akt/protein kinase B (Akt/PKB), Ras-related C3 botulinum toxin substrate 1 (Rac1), the TBC1 domain family member 1 (TBC1D1), or the Akt substrate of 160 kDa (AS160) [32, 110, 111] (Figure 1).

In type 2 diabetes patients, despite a normal amount of GLUT4 transporters [112], insulin fails, in general, to induce adequate insulin signaling as assessed by IRS-1 tyrosine phosphorylation, Akt/PKB activity, and translocation of GLUT4 to the cell membrane [113–116].

Exercise activates the insulin-signaling pathways, facilitating GLUT4 expression and translocation to the cell membrane. The effects of acute and chronic exercise on glucose uptake and insulin signaling are shown in Table 1.

Acute continuous endurance exercise with 45–60 min of training at 65–75% of maximum oxygen consumption (VO_2max_) leads to higher rates of tyrosine phosphorylation of insulin receptor and IRS-1/2 and to increased activity of PI3-K in muscle of untrained healthy as well as insulin-resistant individuals [12–15]. In contrast, short and light resistance with 5 sets of 8 repetitions of isokinetic leg extension shows no effect in endurance trained athletes [16] (Table 2). Furthermore, acute muscle contraction activates molecules of the distal insulin signaling which are known to be involved in GLUT4 translocation such as Rac1, AS160, and TBC1D1 [16, 47, 48] which will be described in more detail below. Recent animal studies have shown that only very intense muscle contraction in situ via sciatic nerve stimulation of multiple muscle types with 2–5 V as well as one bout of intense swimming for 120 min or 60 min of running with a speed of 22 m/min and incline of 10% led to an acute increase in phosphorylation and activity of key molecules like the different AKT isoforms (AKT-1, AKT-2, and AKT-3) and AS160 [49–51] (Table 3).

In contrast to these studies, some human as well as animal studies reported no effect of acute exercise on proximal insulin signaling like changes in insulin receptor amount, IRS-1 phosphorylation, or PI3-K activity [16–21]. In the study of Wojaszewski et al. [21], one-legged cycling exercise for 60 min at intensity of 18–23% of VO_2max_ was not sufficient to induce changes in proximal insulin signaling in young trained individuals. Furthermore, 60 min of cycling at 75%  VO_2max_ did not lead to changes in proximal signaling in untrained and obese individuals [19, 20]. In line with this, some animal studies found that a running speed of 18 m/min for 45 min as well as electrical stimulation with 1–3 V were also not sufficient to induce insulin signaling in skeletal muscle [17, 18].

The reason for these discrepant results in human and animal studies might lie in the differences in the intensity of training conditions in acute exercise. Moderate endurance exercise seems to acutely increase proximal signaling in untrained individuals [12, 13, 15], whereas short and light resistance and endurance training in trained individuals shows no effect [16, 21] (Tables 2 and 3). In addition, the time point after exercise when the effect of exercise is studied appears to be highly important. A recent review from Frøsig and Richter identified a critical time point of 3 to 4 h after exercise for exercise-induced increase in glucose uptake indicating a time-dependent course in the activation of exercise induced molecular signaling [22], which may be the reason for the unaltered signaling in measurements 16 and 24 h after exercise termination [19, 20]. Though training intensity and the time point of investigation appear to be important for exercise-induced activation of insulin signaling, there is still a lack of knowledge about the underlying mechanisms of acute exercise and effects of different training factors, such as modality and intensity, on insulin signaling.

Chronic exercise can also lead to higher rates of tyrosine phosphorylation of key molecules in the insulin signaling cascade in muscle of healthy as well as insulin-resistant individuals [52, 95, 96]. A recent exercise study observed enhanced whole-body insulin action and increased Akt and AS160 phosphorylation after 10 weeks of chronic resistance training with exercises for upper and lower body and running endurance training in untrained individuals [52] indicating an independence of exercise modality. Compared to untrained controls, trained humans show increased insulin-stimulated PI3-kinase activation. The positive association between PI3-kinase activation and endurance capacity (VO_2max_) indicates that regular exercise leads to greater insulin-stimulated IRS-1-associated PI3-kinase activation in human skeletal muscle [14]. This is in line with recent animal studies showing that intense chronic endurance training in mice with a running speed of 20–32 m/min on a treadmill increases total AS160 phosphorylation [53].

### 3.2. AMPK Signaling Pathway

AMPK is a metabolic master switch regulating several intracellular systems and consists of two catalytic alpha-isoforms: *α*2- and *α*1-AMPK. AMPK is activated by phosphorylation by kinases such as liver kinase B1 (LKB1) [117] and is regulated by cellular energy demand. Increasing adenosine monophosphate/adenosine triphosphate (AMP/ATP) and creatine/phosphocreatine (Cr/PCr) ratios, reflecting for instance the glucose deprivation state [118], are important stimuli for AMPK activity. In line with this, activation of AMPK is positively associated with an increased skeletal muscle glucose uptake [23].

In obese diabetic and nondiabetic humans, exercise-induced stimulation of the AMPK activity is attenuated but can be fully activated by exercise with higher intensities of training as compared to healthy lean controls [24, 25]. Acute cycling endurance exercise at a moderate intensity of 50–70% of VO_2max_ increased AMPK activity and resulted in a 2.7-fold increase in mRNA expression of AMPK*α*1 and AMPK*α*2 [24, 25].

Activation of AMPK by acute cycling exercise led to an enhanced glucose uptake in human skeletal muscle [26]. AMPK phosphorylation and activity showed an intensity-dependent response pattern. More intense (80% of VO_2max_) acute cycling endurance exercise with the same amount of energy expenditure (400 kcal) resulted in a higher activation of signal transduction compared to less intense (40%  VO_2max_) endurance exercise [27]. High-intensity interval training consisting of repeated sessions of intense work like all-out sprints for 30 sec (SIT) induces, with a minimum of effort (<80 kJ total), an increased phosphorylation of AMPK. Though this kind of training appears to mimic resistance exercise because of the intense, short-term muscle work, phosphorylation and activity of downstream targets linked to hypertrophy like p70 ribosomal S6 kinase and 4E binding protein 1 were unchanged [28] (Table 2).

While some acute exercise studies in animals showed that AMPK-deficient mice and LKB-deficient mice had a normal contraction-induced glucose uptake, which was independent of the knockout of the catalytic alpha-isoforms of AMPK [97, 98], other studies found that pharmacological inhibition of AMPK and LKB activity blunted contraction-induced glucose disposal in animal models by electrical stimulation [99, 100]. LKB1 knock-out in muscle provoked a reduced activity of the AMPK*α*2 isoform, and transgenic mice expressing a kinase-dead, dominant negative form of the AMPK*α*2 showed also a reduced AMPK activity and blunted glucose uptake. The authors assumed that either the maximal force production was reduced in this muscle, raising the possibility that the defect in glucose transport was due to a secondary decrease in force production and not impaired AMPK*α*2 activity, or the kinase-dead, dominant negative form of the AMPK*α*2 had a negative influence on glucose uptake [99, 100].

Chronic endurance as well as resistance exercise also induces AMPK activation and leads, furthermore, to changes in gene expression favoring GLUT4 translocation. AMPK phosphorylation is more strongly increased after 10 weeks of cycling at 65%–90% of maximum performance (*W*_max_) exercise than after 10 weeks of leg-focused resistance training with an intensity of a 4-5-repetition maximum (RM) [8, 29]. In animal studies, chronic treadmill running as well as resistance training in the form of ladder climbing with weights activated AMPK phosphorylation and up-regulated expression of AMPK in rat pancreatic islets and skeletal muscle [30, 31] indicating that AMPK upregulation is independent of exercise modality in different tissues (Tables 2 and 3).

In light of the animal studies showing that AMPK-deficient mice have a normal contraction-induced glucose uptake [97, 98] other molecular pathways, comprising the Ca^2+^/calmodulin signaling pathway, appear to modulate exercise-induced glucose uptake and will be described in the following subsections.

### 3.3. Ca^2+^/Calmodulin Signaling Pathway

Changes of the calcium concentration in skeletal muscle cells lead to activation of signaling cascades that influence cellular metabolism including glucose uptake [119]. In diabetes, the calcium- (Ca^2+^-) dependent signaling pathway and subsequently glucose uptake are impaired [120]. Genome-wide studies for DNA methylation have shown that first-degree relatives of patients with diabetes have already altered DNA methylation of genes encoding proteins involved in calcium-dependent signaling compared to healthy individuals without positive family history. However, DNA methylation decreased after 6 months of cycling and aerobic exercise [33].

As result of skeletal muscle contraction, cytosolic Ca^2+^ concentration and consequently the number of Ca^2+^/calmodulin complexes increase. Further important key players in the Ca^2+^/calmodulin signaling pathway are the Ca^2+^/calmodulin-dependent protein kinases (CaMKs). These components are critical for exercise-induced glucose uptake [32]. Downstream components of the Ca^2+^/calmodulin signaling pathway are members of the histone deacetylase (HDAC) family and proteins of the myocyte enhancer factor 2 (MEF2) family leading to an enhanced expression rate of GLUT4 [8].

Ca^2+^ release and phosphorylation of CaMKII after acute endurance cycling exercise depend on training intensity. A matched amount of work with different intensities of 40% and 80% of VO_2max_ led to an increase in CaMKII phosphorylation by 84% immediately after high-intensity but not low-intensity cycling endurance exercise indicating that greater force outputs result in enhanced Ca^2+^/calmodulin signaling [27]. Furthermore, also the duration of endurance exercise affects the Ca^2+^/calmodulin signaling pathway activity, with higher activity after longer duration. A 90-min acute cycling endurance exercise resulted in a progressive increase of CaMKII activity during exercise peaking at 90 min of training [34]. In line with this, a recent study comparing acute HIT cycling with traditional continuous cycling exercise showed a marked increase of CaMKII activity by HIT despite the same amount of total work after 30 min of 70%  *W*_max_ [35] (Table 2).

In accordance with the acute exercise studies, a recent animal study showed that chronic endurance training on a treadmill increased the phosphorylation of CAMKII in pancreatic islets of rats in a dose-response manner [31].

In experimental mouse studies, incubation with the Ca^2+^/calmodulin inhibitor KN-93 decreased skeletal muscle glucose transport [121] and inhibited electrical contraction-induced CaMKII phosphorylation [102]. In addition to the decrease of contraction-induced glucose uptake via electrical stimulation, inhibition of CaMKII resulted in an increase of AMPK activity in a recent mice study, pointing to overlapping mechanisms between these two key signaling pathways: the Ca^2+^/calmodulin signaling pathway and the AMPK-signaling pathway [103]. Another key player in the context of glucose uptake-related signaling is the protein kinase mammalian target of rapamycin (mTOR) that will be addressed in the following section.

### 3.4. Mammalian Target of Rapamycin/Serine Kinase 6 (mTOR/p70^SK6^) Pathway

MTOR is a serine/threonine protein kinase that integrates diverse environmental cues by translating them into appropriate cellular responses. Disrupting the mTOR signaling pathway causes a decrease in glucose uptake in multiple cell types such as brain, muscle, and adipose tissue [122–124] and can lead to insulin resistance [125].

High-force stimuli like resistance training lead to muscle adaptation preparing skeletal muscle for more intensive stress. This muscle adaptation which appears to be dysregulated in an insulin-resistant and diabetic state is initiated by the activation of the mTOR/p70^S6K^ pathway [36, 126, 127]. This protein complex activates signaling cascades including binding proteins (elF4E), initiation factors (4E-BP1), and elongation factors (eEF2) leading to protein synthesis and subsequently to cellular hypertrophy [128]. MTOR also stimulates focal adhesion kinases (FAK) and increases FAK-phosphotransferase activity in order to activate muscle protein synthesis [129]. This adaptation is related to the intensity of the muscle contraction, increasing with higher training load. Acute cycling exercise of 70% of VO_2max_ as well as leg-specific strength exercises of 70% of 1-RM increased mTOR phosphorylation. In particular, resistance training leads to higher activation of mTOR signaling compared to traditional endurance exercise despite a huge difference in workload (660 versus 130 kcal) [29, 37–39].

Protein synthesis is regulated, in particular, by contraction-induced activation of the multiprotein complex mTORC1. This protein complex functions as a sensor or control unit which regulates the translation of proteins by assessing the cellular environment for optimal conditions and initiating translation of mRNA. Besides physical activity, potent stimulators of the mTOR/S6K pathway are insulin, insulin-like growth factor (IGF-1), cytokines like IL-6, sufficient amino acid levels in skeletal muscle, and full-energy depots [130].

During acute endurance as well as resistance exercise, mTOR signaling is inhibited via AMPK phosphorylation and signaling to suppress high-energy demanding procedures such as protein synthesis [40–42]. However, after exercise, muscle protein synthesis increases in parallel to the activation of Akt/PKB (protein kinase B), mTOR, S6K, and eEF2.

One bout of intense treadmill walking at 70% of HR_max_ for 45 min in untrained old men as well as 70% of VO_2max_ of one leg exercise for 60 min in untrained healthy young men led to significant activation of the insulin signaling as well as of the mTOR/SK6 pathway [43, 44]. In line with this, recent exercise studies showed that acute cycling-based HIT or intense leg-specific strength training [40] activates the mTOR signaling pathway in human muscle [39, 45]. Exercise-induced activation of mTOR signaling in leg-specific endurance and resistance training appears to be time-dependent with a continuous increase after termination of physical activity [40, 43]. In line with the human studies, mTOR signaling was upregulated in acute exercise studies in animals comprising treadmill running and electrical stimulation, with a time-dependent answer after exercise termination [46] (Tables 2 and 3).

Chronic exercise studies also demonstrate that long-term leg-specific resistance training with 4-5-RM in sedentary individuals and high intensity cycling with 70–85% of HR_max_ in untrained controls can activate the mTOR signaling pathway in human muscle [29, 38]. These results underline that the activation of mTOR signaling may be independent of exercise type as well as training history. Besides mTOR, there are other important downstream targets modulating glucose uptake that will be addressed in the following section.

### 3.5. Ras-Related C3 Botulinum Toxin Substrate 1 (Rac1), TBC1 Domain Family Members 1 and 2 (TBC1D1/2), and Akt Substrate of 160 kDa (AS160)

The proteins AS160, TBC1D1/2, and Rac1 are involved in insulin- as well as contraction-induced glucose uptake [131, 132] and are, therefore, points of convergence of these two pathways. These downstream targets are altered in an insulin-resistant or diabetic state showing a reduced signaling activity [101, 133–136].

Acute endurance exercise studies in untrained and trained humans showed an increase in phosphorylation of TDC1D1/4 and AS160 in skeletal muscle in the first 4 hours after cycling and specific one-leg endurance exercise, especially under long-term training conditions with a training duration of at least 60 min at 65%  VO_2max_ [16, 54–56]. In line with these human studies, animal studies found that contraction-induced glucose uptake by electrical stimulation was also modulated by an increase in phosphorylation of AS160 and TBC1D1 proteins [57] (Tables 2 and 3).

Rac1, a key downstream target in the regulation of glucose uptake, was shown to modulate exercise- and insulin-stimulated GLUT4 translocation in human muscle, with an intensity-dependent response pattern, as shown in murine muscle [58, 59]. Animals were exercised at their 50% and 70% maximum running speed over 30 min on a treadmill, and the higher intensity program resulted in an larger increase of Rac1 activation. Given that the total amount of work differed between both measurements, the results are hard to interpret. The larger improvement may result from the higher intensity or from the greater amount of exercise. A future study comprising an alternative training protocol with identical energy expenditure but different intensities would help to clarify the role of exercise intensity in this context. Furthermore, in RAC1-deficient mice, GLUT4 translocation as well as glucose uptake decreased after acute electrical stimulated muscle contraction and insulin infusion as a sign of an inhibited signaling capacity [59, 60].

Glucose uptake and insulin signaling are influenced by inflammatory processes and specific cytokines [2]. The following section aims at shedding some light on the impact of inflammatory signaling on exercise-stimulated glucose uptake and insulin signaling.

## 4. Inflammation-Associated Signaling Pathways and Key Players

### 4.1. I*κ*B Kinase/Nuclear Factor Kappa B Pathway (IKK/NF-*κ*B)

Different environmental influences, for example, certain pathogens, can activate molecular signaling cascades leading to an inflammatory response mediated by the IKK/NF-*κ*B pathway. Recognizing receptors are, in particular, Toll-like receptors (TLRs). TLR4 plays a key role in the activation of the pro-inflammatory NF-*κ*B pathway. TLRs interact with pathogen-associated molecules, resulting in an activation of downstream signaling proteins, for example, MyD88 [137], and subsequently an immune reaction via cytokine release, for example, of IL-6 and TNF-*α* from adipose tissue. The adapter protein MyD88 also activates other inflammation-associated signaling pathways like MAPK signaling as described below in more detail [138]. TLRs are expressed on macrophages, which can be subdivided into pro-inflammatory M1 and anti-inflammatory M2 macrophages. Exercise studies have shown that physical activity modulates TLR-dependent pathways [2]. As a result, acute as well as chronic exercise can lead to reduced TLR expression [61] and phenotypic switching from M1 to M2 macrophages in adipose tissue of obese mice [62].

Cytokines like IL-6 or agents comprising microbial components trigger signaling cascades that converge in the activation of I*κ*B kinase (IKK) enzyme complex and subsequently in a translocation of the protein complex NF-*κ*B into the nucleus. This results in transcription of target genes for inflammatory immune reaction including cytokines like IL-6, TNF-*α*, and IL-15 [139]. Chronic activation of the NF-*κ*B pathway contributes to insulin resistance and muscle wasting. Especially in type 2 diabetes, human muscle is characterized by an increased activity of this pathway [63].

Human and animal exercise studies have shown that acute as well as chronic exercise can reduce the activation of the IKK/NF-*κ*B pathway. This attenuation of the inflammatory signaling was independent of the exercise modality, age, and training status [63–69] (Tables 4 and 5).

During acute physical activity with a sufficient load, muscle contraction induces a marked increase of IL-6 expression in skeletal muscle but also suppresses IL-6 production in adipose tissue [70]. Increasing energy demands due to prolonged or intense acute training like marathon running or cycling at 88% of VO_2max_ [71–73] as well as shrinking depots of muscle glycogen [104] accelerate the increase of IL-6 plasma levels. Interestingly, a recent work from Castellani et al. showed that exercise induces also a specific increase of IL-6 in adipose tissue which occurred more rapidly in adipose tissue from trained mice in comparison to untrained mice when exercised at the same relative running speed on a treadmill. The authors speculated that the increase of IL-6 would be needed for the provision of lipids to the muscle and liver [74]. In line with this, Macpherson et al. showed an increasing IL-6 and decreasing M1 macrophages content in inguinal adipose tissue and an improved insulin action after an acute bout of treadmill running exercise in obese mice [75]. In line with the results of the acute exercises studies, chronic exercise also led to decreased activity of the IKK/NF-*κ*B pathway after 8 weeks of cycling exercise at 70% of VO_2max_ and intense whole-body strength exercise with 50–80% of 1-RM [63, 66] (Tables 4 and 5). Accordingly, a decreased plasma IL-6 concentration at rest as well as in response to chronic exercise appears to characterize a normal training adaptation [71].

The transient rise in IL-6 also appears to be responsible for the production of anti-inflammatory mediators like IL-10 or IL-1 receptor antagonist (IL-1RA). In particular IL-1RA prevents inflammatory processes by blocking signal transduction of the proinflammatory IL-1 and creates also an anti-inflammatory balance to the proinflammatory cytokine IL-1*β* [76, 77, 140]. Furthermore, elevated levels of IL-6 from skeletal muscle stimulate an anti-inflammatory signaling cascade that inhibits the secretion of proinflammatory cytokines like TNF-*α* or IL-1*β*, suppress the secretion of the acute-phase reactant C-reactive protein (CRP) from the liver, a general and unspecific marker for systemic inflammation [76, 77], downregulate monocyte TLR expression at both mRNA and cell surface protein levels, and finally inhibit the IKK/NF-*κ*B pathway [64–66]. Besides the TLR family, there are other receptor proteins like NOD-like receptors initiating inflammatory processes and subsequently modulating glucose uptake-related signaling which will be discussed in the following section.

### 4.2. Inflammasome Pathway

The NOD-like receptor (NLR) family is of key importance in the innate immune system. NLRs are responsible for recognizing pathogen and danger-associated molecular patterns. In response to stress signals, NLRs activate the inflammasome pathway which forms a multi-protein complex [2]. Participating components of inflammasome complexes are NLRs, neutrophilic alkaline phosphatases (NALPs), apoptosis-associated speck-like protein (ASC) and caspase-1. After its formation, this oligomer converts proinflammatory cytokines into active forms such as IL-1*β*. Increasing IL-1-*β* levels have been hypothesized to play a role in the progression of type 2 diabetes and its complications because its activity stimulates inflammatory processes leading to cell damage and apoptosis, in particular in pancreatic *β*-cells. Furthermore, IL-1*β* inhibits proximal and distal insulin signaling and mediates interorgan cross talk between adipocytes and the liver, contributing to systemic inflammation [2, 141–143].

A recent review reported that chronic endurance and resistance training in mice decrease NLR family pyrin domain containing 3 (NLRP3) mRNA levels accompanied by reduced IL-18 levels, reflecting diminished activity of the NLR/inflammasome pathway [2]. IL-18 expression decreases under chronic intense endurance exercise conditions with sports like rowing, running, or cycling with an intensity which is at 70% of VO_2max_ in humans [78, 79]. Only chronic training conditions, but not acute exercise, appear to reduce IL-18 mRNA expression [79]. In line with this, a recently published animal study with chronic treadmill running as endurance exercise and isometric strength training as resistance training showed a decrease of IL-18 expression in adipose tissue and plasma levels [80] (Table 5).

So far, there are no human exercise studies which measured acute or chronic effects of physical activity on the upstream elements of the inflammasome pathway. Further mechanistic studies are, therefore, needed to better understand the role of the inflammasome in the anti-inflammatory response to exercise. In contrast, the role of the C-Jun N-terminal kinase (JNK)/mitogen-activated protein kinase (MAPK) pathway in the modulation of exercise-dependent effects on glucose uptake and inflammatory response has been investigated by several animal as well as human studies.

### 4.3. C-Jun N-Terminal Kinase (JNK)/Mitogen-Activated Protein Kinase (MAPK) Pathway

Lipid accumulation in adipocytes and endoplasmic reticulum (ER) stress as well as a NF-*κ*B dependent cytokine releases activate the JNK/MAPK pathway [139, 144]. This activation results in the serine phosphorylation of IRS-1 and the phosphorylation of the c-Jun component of activator protein-1 (AP-1). The phosphorylation of serine residues in insulin receptor substrate-1 leads to an impairment in the ability of IRS-1 to activate downstream phosphatidylinositol 3-kinase-dependent pathways which may cause insulin resistance [145–147]. AP-1 is a transcription factor that mediates the gene expression of many cytokines. Subsequently, the JNK pathway leads to an inflammatory reaction, especially to TNF-*α* and IL-6 release [139]. JNKs are divided into 3 isoforms and belong to the MAPK family. The MAPK family comprises extracellular regulated kinases (ERKs), JNKs and p38, and mediates cell growth, differentiation, hypertrophy, apoptosis, and inflammation [144]. Furthermore, oxidative stress following reactive oxygen species (ROS) production induces JNKs and p38 MAPK activation reflecting an important immune defense mechanism [148]. JNK activation by skeletal muscle contraction is also associated with an increase in muscle IL-6 mRNA expression in mice acutely after endurance exercise in form of treadmill running [81].

Exercise studies in human and animal models showed that the JNK/MAPK pathway is activated in a dose-response pattern. In particular very intense acute exercise like marathon running or cycling at 70% of VO_2max_ and intense dynamic pull exercise as resistance training with an one-repetition maximum (1-RM) of 85% stimulate JNK signaling in skeletal muscle [82–84], independently of training modality. JNK activation results, as a physiological mechanism, in DNA repair and muscle regeneration [149]. In contrast, a recent animal study has shown that acute long-term exercise by swimming for 180 min reduces JNK phosphorylation and improves insulin signaling and sensitivity in adipose tissue from obese rat [69]. In particular, chronic endurance exercise in form of swimming and treadmill running contributes to a reduction in JNK phosphorylation and improves insulin signaling and sensitivity in adipose and hepatic tissue from obese rats [67–69, 85].

Inflammatory signaling pathways are associated with insulin resistance and impaired glucose uptake, whereas adiponectin is an important, though controversially discussed, counterpart being positively associated with insulin sensitivity. This adipokine will be discussed in the following section.

### 4.4. Adiponectin

Adiponectin, an adipokine which is primarily released by white adipose tissue (WAT), appears to be a key player in glucose metabolism at least in rodents, whereas its relevance in humans is somewhat less clear [6]. The secreted adiponectin binds to its receptors AdipoR1 and AdipoR2 and activates AMPK, p38 MAPK, and peroxisome proliferator-activated receptor *α* (PPAR-*α*) following adaptor protein 1 (APPL1) release in skeletal muscle and liver [150]. As a result, adiponectin positively affects metabolism by increasing fatty acid oxidation and glucose uptake in muscle. Furthermore, it plays a critical role in the cross talk between different insulin-sensitive tissues [151, 152]. Adiponectin levels are decreased in patients with diabetes and low adiponectin levels are associated with insulin resistance and obesity [153, 154]. Recent mouse studies showed that pharmacological adiponectin agonists improve insulin sensitivity and other health-related parameters [155].

Only a limited number of acute exercise intervention studies focused on changes of adiponectin levels. In one study, circulating adiponectin levels increased 30 min after endurance exercise in the recovery phase [5]. The currently available data indicate that adiponectin levels change in dependence of exercise intensity, showing an increasing level by enhanced training intensity of 76%  VO_2max_ in trained rowing athletes [86], whereas moderate and long-lasting cycling at 50% of VO_2max_ for 120 min did not acutely increase adiponectin levels in trained individuals immediately after exercise [87] (Table 4).

Conflicting results were also observed under chronic exercise conditions. More intense endurance exercise in form of cycling and brisk walking at 70% of VO_2max_ resulted in increases of adiponectin levels [88, 89]. Overweight and age seem to reduce the response of adiponectin to exercise [90]. In line with this, Simpson and Singh reported in their review that adiponectin expression levels are increased under high-intensity exercise conditions [91], regardless of training modality in untrained young lean or obese individuals, after chronic whole-body strength training or jogging [92, 93]. In line with this, Cho et al. showed that 40 minutes of HIT exercise on treadmill prevent the downregulation of AdipoR1 which was caused by a high fat diet in sedentary control animals [94] indicating the importance of intense training for the potential role of adiponectin.

In contrast, untrained and trained adiponectin knockout mice (AdKO) significantly increased glucose tolerance and insulin sensitivity after 8 weeks of treadmill running suggesting the presence of an unknown compensatory mechanism [53].

A recent meta-analysis found that chronic exercise did not significantly increase adiponectin levels. However, in subgroup analyses, all modalities tended to increase adiponectin. The lack of statistical power due to small group sizes may have contributed to the overall null-finding [3]. In contrast, lifestyle interventions with unstructured exercise alone or in combination with weight-reducing diet can positively influence adiponectin plasma levels [156]. Weight loss is an important factor contributing to increases in plasma levels of adiponectin [157–159]. In conclusion, the impact of exercise on adiponectin levels needs further clarification. With respect to chronic effects it is important to investigate to what extent exercise effects on adiponectin may be mediated by weight loss.

### 4.5. Exercise, Inflammation, and Insulin Signaling

Circulating serum or plasma levels of cytokines are strongly linked with the onset of type 2 diabetes [160–162]. The stimulation of inflammatory signaling cascades can lead to interference with the insulin signaling pathway [2]. During exercise, acute effects on cytokine regulation comprise an upregulation of both (i) proinflammatory cytokines (e.g., TNF-*α*, IL-1*β*, and IL-6) and (ii) anti-inflammatory cytokines (IL-1RA, IL-10) [106].

Long-term effects of physical exercise are known to reduce markers of inflammation by decreasing adipocytokine production and cytokine release from skeletal muscle [163–165]. The relationship between glucose uptake and adiponectin, IL-6, and TNF-*α* is shown in Figure 1.

The mechanistic impact of inflammation on insulin signaling has been studied for several cytokines. Currently available data suggest that TNF-*α* plays a direct role in the development of insulin resistance by decreasing glucose uptake into adipocytes via suppression of insulin receptor activity, AMPK activation, and downregulation of GLUT4 expression [165–168]. Acute exercise did not change the expression pattern of TNF-*α* [169], whereas the increase of TNF-*α* during high intense physical activity like marathon running appears to be a response to muscle damage [104, 107, 108, 170]. Large cohort studies show that physical activity or chronic endurance exercise in form of walking reduces systemic subclinical inflammation [92] and the impact of exercise rises in a dose-response pattern regulated by frequency and intensity, but inflammation remains unchanged when exercise intensity was only moderate [66, 109, 171, 172]. A moderate community-based walking program with 3000 steps more per day did not change TNF-*α* plasma levels [109] and a chronic resistance training with only 2 units per week of only 3 sets of 3 exercises had also no impact on TNF-*α* protein content. In line with this, TNF-*α* plasma levels were reduced by high-intensity chronic resistance training, even though fat mass has not changed [173]. Also animal studies show that chronic exercise training, in particular endurance training like treadmill running, can reduce TNF-*α* levels [62].

IL-6 is another important protein in this context and is expressed by several tissues. As a myokine, muscle-derived IL-6 is acutely upregulated during exercise exposure [106] and mediates a physiological cross talk with WAT and liver in order to regulate glucose metabolism [160]. However, long-term effects of regular exercise show marked decreases of IL-6 levels [77]. The role of IL-6 is complex, as also evident by its diverse effects on molecular signaling. In adipose tissue, IL-6 mediates inflammatory processes and causes insulin resistance by downregulating GLUT4 and IRS-1 expression [139]. Furthermore, increasing IL-6 levels block PI3-K, another key player in insulin signaling, and induce TLR4 gene expression leading to enhanced inflammatory processes [174, 175]. In addition, IL-6 induces the downstream NF-*κ*B signaling pathway which impairs insulin signaling and subsequently induces insulin resistance in insulin-dependent tissues of obese humans and animals [2]. In particular, IL-6 and liver interact in the context of exercise. A human exercise study with long-term cycling has shown that contraction-induced IL-6 release increased endogenous glucose production (EGP), thus underlining the importance of IL-6 for glucose homeostasis [105].

Experimental studies using mouse models yielded controversial findings. IL-6-deficient mice can develop a glucose-intolerant and insulin-resistant state indicating that balanced IL-6 levels have a positive effect on glucose uptake. Furthermore, mouse studies showed that circulating IL-6 levels increase glucose uptake and improve insulin sensitivity in skeletal muscle via AMPK activation [176].

Besides, the inflammasome pathway downregulates insulin signaling. The inflammasome pathway which is part of the innate immune system converts proinflammatory cytokines into active forms such as IL-1*β* or IL-18 which are decreased in their levels after chronic exercise [2]. Important proinflammatory chemokines which are influenced by exercise are interleukin 8 (IL-8) and monocyte chemoattractant protein-1 (MCP-1). Both cytokines slightly increase after acute exercise; however, their circulating levels decrease after chronic exercise in human as well as animal model exhibiting an improved inflammation status [75, 77, 139, 160], as shown in Table 1.

## 5. Summary

Exercise is an important cornerstone in the prevention and treatment of metabolic disorders. Acute and chronic exercise activates different molecular signaling pathways that can counteract defects in signaling and associated metabolic processes (Table 1). Exercise interventions have shown that physical activity can increase GLUT4 protein expression and translation by activation of different molecular signaling pathways irrespective of the exercise modality. AMPK and Ca^2+^/calmodulin signaling pathways show a dose-response pattern and increase their activity with increasing intensity despite equal work rate in kcal when compared to less intense exercise.

The key players mTOR, AS160, TBC1D1/4, and Rac1 can be activated by exercise. Human exercise studies have demonstrated that acute and chronic physical activity, regardless of training modality, leads to increases in their activity and finally to improved glucose uptake. The change in activity reflects a dose-response pattern. MTOR and AS160 also exhibit a continuous time-dependent increase.

Metabolic disorders are accompanied by activated inflammation-related signaling pathways which result in elevated cytokine release. Proinflammatory immune mediators, like IL-1*β*, IL-6, or TNF-*α*, are important factors in the development of insulin resistance. Their expression is modulated by physical activity. In particular, chronic endurance and resistance training and high training intensity improve glucose uptake which is associated in the long term with decreased secretion of proinflammatory cytokines and increased release of anti-inflammatory proteins such as adiponectin.

In summary, the current literature points to a higher efficiency of more intense exercise because of a dose-response relationship regulating metabolic improvements. However, more high-quality exercise interventions as well as mechanistic studies have to be performed to fully understand the molecular mechanisms contributing to metabolic improvements.

## 6. Open Questions

Despite the high number of studies on exercise interventions and underlying mechanisms that have been conducted, we are far from understanding the details mediating the effects of exercise on glucose uptake. Single key players in this field were identified over time and confirmed with mechanistic human and animal studies. So far, there is still a lack of knowledge about the underlying mechanisms of exercise-induced glucose uptake in regard to training factors, such as point of termination or intensity, especially in proximal insulin signaling. When interpreting the responses to training, it is important to know, in particular when dealing with the issue of glucose uptake and related signaling pathways, when relative to the last bout, and preferably the last two bouts, the samples were collected to distinguish between acute and chronic training effects. Furthermore, exercise exposure can be considered the combined responses to intensity, bout duration, and bout frequency, where the product is usually considered to be total amount like total energy expenditure, but only a small number of intervention studies controlled for total work.

In regard to the key players of molecular signaling, the interplay of interacting pathways, such as the Ca^2+^/calmodulin signaling pathway and the AMPK pathway, is still elusive. The inflammatory signaling pathways involving IKK/NF-*κ*B and the inflammasomes have not been sufficiently characterized in the context of the influence of acute as well as chronic exercise. The controversial results of the adiponectin exercise studies highlight potential species differences between men and mice and merit more mechanistic studies.

Furthermore, there is an intense need to detect to what extent the effects of physical exercise are independent of or explained by weight loss or change in body composition. Some of these questions require larger sample sizes and higher statistical power to quantify effects but also standardized methods for molecular measurements and high-quality study plans considering potential confounders.

## Figures and Tables

**Table 1:** Effect of acute and chronic exercise on molecular signaling pathways.

Metabolic factor	Acute training	Chronic training	Exercise characteristics (intensity, modality)	References
Proximal insulin signaling (IRS-1, PI3-K, PDK, *α*PKC)	↑^*∗*^	↑↑	Moderate-to-intensive exercise for untrained and high-intensity exercise for trained individuals, independent of modality	[12–22]

AMPK	↑↑	↑↑	Dose-response pattern, independent of modality	[8, 23–31]

Ca^2+^-calmodulin axis	↑↑	↑↑	Dose-response pattern, independent of modality	[8, 27, 31–35]

mTOR/S6K	↑↑	↑↑	Dose-response pattern, independent of modality	[29, 36–46]

Downstream targets: AS160, TBC1D1, Rac1	↑	↑	Dose-response pattern for AS160 and Rac1, independent of modality	[16, 47–60]

IKK/NF-*κ*B pathway	↑↕^$^	↓↓	Dose-response pattern, independent of modality	[2, 61–77]

Inflammasome pathway	—	↓↓	Dose-response pattern, independent of modality	[2, 78–80]

JNK/MAPK pathway	↑↑	↓↓	Dose-response pattern, independent of modality	[67–69, 81–85]

Adiponectin	↑	↑	Intense exercise, independent of modality	[3, 5, 53, 86–94]

↑↑/↓↓, consistent findings in animal models and humans; ↑/↓, preliminary evidence from animal models and/or humans; —, no impact; ^*∗*^animal studies showed no effects; ^$^increase in skeletal muscle and increase/decrease in adipose tissue; *α*PKC, atypical PKC; AMPK, AMP-activated protein kinase; AS160, Akt substrate of 160 kDa; Ca, calcium; CaMKII, Ca^2+^/calmodulin-dependent protein kinase 2; IRS-1, insulin receptor substrate 1; IKK/NF-*κ*B, I*κ*B kinase/nuclear factor kappa B; JNK, C-Jun N-terminal kinase; MAPK, mitogen-activated protein kinases; mTOR/S6K, mammalian target of rapamycin/ribosomal S6 kinase; PDK, phosphoinositide-dependent kinase; PI3-K, phosphoinositide 3-kinase; Rac1, ras-related C3 botulinum toxin substrate 1; TBC1D1, TBC1 domain family member 1.

**Table 2:** Influence of exercise on glucose uptake-related signaling pathways in humans.

Reference	Study population, *n*	Age, years	Training modality	Type of sport	Training frequency	Acute/chronic exercise	Training intensity	Tissue & condition	Time since the last exercise bout, h	Changes in glucose uptake and related molecular signaling^*∗*^
Cusi et al., 2000 [12]	9 untrained obese CON, 10 untrained T2D	44 ± 4 42 ± 3	ET	Cycling	60 min	Acute	65% VO_2max_	Muscle & insulin-stimulated (clamp)	24 h after exercise	Increase of insulin receptor (+60% in obese CON, +34% in T2D) and IRS-1 tyrosine phosphorylation (+20% in T2D)

Howlett et al., 2006 [15]	7 untrained CON	24 ± 2	ET	Cycling	60 min	Acute	75% VO_2max_	Muscle & insulin-stimulated (clamp)	Immediately after exercise, and at 30 and 120 minutes of clamp	Increase of insulin-stimulated IRS-2 signaling (IRS-2-associated PI3–kinase activity) after exercise

Perseghin et al., 1996 [13]	10 untrained lean offspring T2D, 8 untrained CON	33 ± 3 29 ± 2	ET	Stair-climbing machine	45 min	Acute	65% VO_2max_	Muscle & insulin-stimulated (clamp)	48 h after exercise	Increase of glucose disposal by 35% in the offspring and 41% in CON

Wojtaszewski et al., 2000 [21]	7 trained CON	22 ± 1	ET	One-leg-exercise	60 min	Acute	18–23% VO_2max_	Muscle & insulin-stimulated (clamp)	After 7, 15, 60, 120, 150 min of exercise	No change in proximal insulin signaling, but exercise induced increase of glucose uptake up to 2-to-4-fold higher compared to rested leg

Musi et al., 2001 [24]	7 untrained lean T2D, 8 untrained CON	53 ± 3 49 ± 1	ET	Cycling	45 min	Acute	70% of *W*_max_	Muscle	During and immediately after exercise	Similar protein expression of AMPK *α*1, *α*2, and *β*1 in muscle of T2D, compared with CON, increase of AMPK*α*2 activity (2.7-fold) after exercise

Gibala et al., 2009 [28]	6 trained CON	23 ± 2	HIT	Cycling	20 min	Acute	4 × 30 s “all-out” sprint	Muscle	Immediately and 3 h after exercise	Increase of AMPK (30%), AMPK*α*1 (20%), and AMPK*α*2 (80%) phosphorylation

Sriwijitkamol et al., 2007 [25]	8 CON, 8 obese CON, 12 T2D	45 ± 3 44 ± 4 53 ± 3	ET	Cycling	40 min	Acute	50–70% VO_2max_	Muscle	During and immediately after exercise	AMPK activity only improved in lean CON in a dose-response manner

Benziane et al., 2008 [26]	9 untrained CON	23 ± 2	ET	Cycling	60 min	Acute	164 W (intense)	Muscle	Immediately and 3 h after exercise	Increase of AMPK (16.0-fold) and mTOR (2.0-fold) phosphorylation after exercise and abrogation of AMPK phosphorylation and mTOR phosphorylation after 3 h of exercise

Egan et al., 2010 [27]	8 sedentary CON	25 ± 1	ET	Cycling	n.r.	Acute	40/80% VO_2max_ (400 kcal)	Muscle	Immediately, 3 h and 19 h after exercise	Increase of AMPK (2.8-fold) and CaMKII (84%) phosphorylation immediately after high-intensity but not low-intensity exercise

Rose et al., 2006 [34]	8 trained CON	25 ± 1	ET	Cycling	90 min	Acute	67% VO_2max_	Muscle	At rest and after 1, 10, 30, 60, and 90 min of exercise	Increase of CaMKII activity during exercise depending on exercise duration (2-fold)

Rose et al., 2006 [34]	10 trained CON	25 ± 2	ET	Cycling	30 min	Acute	35%, 60%, 85% VO_2max_	Muscle	Immediately and 30 min after exercise	Increase of CaMKII phosphorylation during exercise depending on exercise intensity (1 to 3-fold)

Combes et al., 2015 [35]	9 trained CON	22 ± 5	ET/HIT	Cycling	30 min/30 × 1 min	Acute	70% of *W*_max_	Muscle	Immediately and 3 h after exercise	Increase of CaMKII phosphorylation by 2.7-fold after HIT compared to continuous exercise (same work rate)

Fujita et al., 2007 [44]	6 untrained CON	70 ± 2	ET	Treadmill walking	45 min	Acute	70% of HR_max_	Muscle & insulin-stimulated (clamp)	20 h after exercise	Increase of mTor activity (5.0-fold) after 20 h of rest under insulin stimulation

Camera et al., 2010 [39]	8 trained CON	29 ± 2	ET	Cycling	60 min	Acute	70% VO_2max_	Muscle	Immediately, 15, 30, and 60 min after exercise	Increase of mTOR phosphorylation (100%) that peaked 30–60 min after exercise termination, workload (660 kcal)

Camera et al., 2010 [39]	8 trained CON	28 ± 2	RT	Leg extension	8 × 5 repetitions	Acute	80% 1-RM	Muscle	Immediately, 15, 30, and 60 min after exercise	Increase of mTOR phosphorylation (100%) that peaked 30–60 min after exercise termination, workload (130 kcal)

Mascher et al., 2011 [43]	16 untrained CON	23 ± 2 25 ± 1	ET	One-leg cycling	60 min	Acute	65–70% VO_2max_ of one leg	Muscle	Immediately, 90 and 180 min after exercise	Time-dependent increase of mTOR phosphorylation after 180 min of recovery by 60% compared to resting situation

Pugh et al., 2015 [45]	10 untrained CON	21 ± 1	RT	Leg extension	4 × 8 repetitions	Acute	70% 1-RM	Muscle	2 h and 6 h after exercise	No change of mTOR after RT alone

Pugh et al., 2015 [45]	10 untrained CON	21 ± 1	RT + HIT	Leg extension + cycling	4 × 8 repetitions + 20 min	Acute	70% 1-RM + 10 times 1 min 90% HR_max_	Muscle	2 h and 6 h after exercise	RT + HIT: increase of mTOR phosphorylation by 30% compared to resistance training alone

Dreyer et al., 2006 [40]	11 untrained CON	27 ± 2	RT	Leg extension	10 × 10 repetitions	Acute	70% 1-RM	Muscle	During and 2 h after exercise	Increase of AMPK phosphorylation (50%) until 1 h after exercise and progressive increase of mTOR phosphorylation up to 100% at 2 h after exercise

Deshmukh et al., 2006 [16]	9 trained CON	29 ± 6	ET	Cycling	60 min	Acute	70% VO_2max_	Muscle	Immediately after exercise	Increase of Akt (80%) and AS160 (100%) phosphorylation in endurance trained young athletes after exercise

Deshmukh et al., 2006 [16]	9 trained CON	29 ± 6	RT	Isokinetic leg extension	8 × 5 repetitions	Acute	Maximal voluntary isokinetic leg extensions	Muscle	Immediately after exercise	No change of Akt and AS160 in endurance trained young athletes after exercise

Treebak et al., 2007 [55]	30 trained CON	26 ± 1	ET	Cycling	20 min, 2 min, 30 sec	Acute	222 W 376 W 666 W	Muscle	Immediately after exercise	No change in AS160 phosphorylation in all 3 study arms

Treebak et al., 2007 [55]	8 trained CON	25 ± 1	ET	Cycling	90 min	Acute	67% VO_2max_	Muscle	Immediately after exercise	Increase of AS160 phosphorylation (120%)

Treebak et al., 2009 [56]	12 trained CON	26 ± 1	ET	One-leg-exercise	60 min	Acute	80% of *W*_max_	Muscle	4 h after exercise	Increase of AS160 phosphorylation in exercised leg by 20–40%

Sylow et al., 2014 [58]	9 CON	n.r.	ET	Inclined walking	45 min	Acute	69% VO_2max_	Muscle	Immediately after exercise	Increase of Rac1 activity by 38% in m. soleus and 52% in m. gastrocnemius; increase of p-Rac1-Ser71 phosphorylation by 39% in m. soleus and by 20% in m. gastrocnemius

Vendelbo et al., 2014 [54]	8 trained CON	26 ± 4	ET	Cycling	60 min	Acute	65% VO_2max_	Muscle	30 min and 4 h after exercise	Increase of AS160 and TBC1D1 phosphorylation 30 min after exercise

O'Gorman et al., 2006 [19]	7 obese CON, 8 obese T2D	48 ± 4 45 ± 2	ET	Cycling	60 min	Acute short term (7 days)	75% VO_2max_	Muscle & insulin-stimulated (clamp)	16 h after exercise	Increase of glucose disposal by 36% in T2D, but not CON, no change in proximal signaling

Wadley et al., 2007 [20]	8 untrained CON	24 ± 1	ET	Cycling	60 min	Acute short-term (7 days)	75% VO_2max_	Muscle & insulin-stimulated (clamp)	24 h after exercise	No change of insulin receptor & IRS-1 tyrosine phosphorylation after either acute or short-term training

Frøsig et al., 2007 [95]	8 trained CON	25 ± 1	ET	One-legged knee extensor apparatus	60–120 min	Short-term (21 days)	70–85% peak work load	Muscle & insulin-stimulated (clamp)	Immediately, 10 and 120 min under insulin after exercise	Increase of Akt1/2 and AS160 protein content by 55% and 25%, but, under insulin stimulation, no exercise effect

Perseghin et al., 1996 [13]	10 untrained lean offspring T2D, 8 untrained CON	33 ± 3 29 ± 2	ET	Stair-climbing machine	4 × 45 min	Chronic (6 weeks)	65% VO_2max_	Muscle & insulin-stimulated (clamp)	48 h after exercise	Increase of glucose uptake by 76% in offspring and 58% in CON

Holten et al., 2004 [96]	10 untrained overweight T2D, 7 untrained CON	62 ± 3 61 ± 3	RT	Leg training program	3 × 30 min	Chronic (6 weeks)	50% 1-RM - 70–80% 1-RM	Muscle & insulin-stimulated (clamp)	16 h after exercise	40% increase in GLUT4 protein content in T2D, no change in CON; increase of protein content of insulin receptor by 19% (CON) and 21% (T2D), increase of PKB-*α*/*β* (Akt1/2) protein content by 22% (CON) and 12% (T2D)

Consitt et al., 2013 [52]	21 sedentary CON	18–84	ET	Running	3 × 60 min	Chronic (10 weeks)	75% VO_2max_	Muscle & insulin-stimulated (clamp)	40 h after exercise	Increase of whole-body insulin action and insulin-stimulated AS160 phosphorylation after exercise by 60% in young and 75% in insulin resistant CON

Consitt et al., 2013 [52]	22 sedentary CON	20–82	RT	Upper and lower body	3 × 45 min	Chronic (10 weeks)	12-RM	Muscle & insulin-stimulated (clamp)	40 h after exercise	Increase of whole-body insulin action and insulin-stimulated AS160 phosphorylation after exercise by 75% in young & old individuals

Vissing et al., 2013 [29]	24 untrained CON	23 ± 1	ET/HIT	Cycling	3 × 40 min	Chronic (10 weeks)	65%–90% of *W*_max_	Muscle	Immediately, 15, 30, 60, and 120 min after exercise	Increase of AMPK phosphorylation by 44% after ET

Vissing et al., 2013 [29]	24 untrained CON	23 ± 1	RT	3 leg-exercises	3 × 8 × 5 repetitions	Chronic (10 weeks)	4-5-RM	Muscle	Immediately, 15, 30, 60, and 120 min after exercise	Increase of AMPK phosphorylation by 10% and increase of mTOR/p70SK6 phosphorylation after 2 h up to 22 h by 91%–281%

Nitert et al., 2012 [33]	13 sedentary CON (positive family history (FH+))	37 ± 4	ET	Cycling/aerobic exercise	3 × 60 min	Chronic (26 weeks)	n.r.	Muscle	48 h after exercise	Decrease of DNA methylation of genes of calcium signaling pathway after exercise in individuals with FH+

Stuart et al., 2010 [38]	6 sedentary CON	37 ± 3	ET	Cycling	30–70 min	Chronic (6 weeks)	70%–85% of HR_max_	Muscle	40–48 h after exercise	Increase of GLUT4 by 66% and phosphor-mTOR by 83%

Data are given as mean ± SD for age; ^*∗*^all changes given in the table were statistically significant; 1-RM, one repetition maximum; AMPK, AMP-activated protein kinase; AS160, Akt substrate of 160 kDa; Ca, calcium; CaMKII, Ca^2+^/calmodulin-dependent protein kinase 2; CON, controls; ET, endurance training; HIT, high-intensity interval training, HR_max_ maximum heart rate, IRS-1/2, insulin receptor substrate 1/2; mTOR, mammalian target of rapamycin (C1 complex 1 & C2 complex 2); n.r., not reported; PDK, phosphoinositide-dependent kinase; PI3-K, phosphoinositide 3-kinase; PKB, protein kinase B; Rac1, ras-related C3 botulinum toxin substrate 1; RM, repetition maximum; RT, resistance training; T2D, type 2 diabetes; TBC1D1, TBC1 domain family member 1; VO_2max_, maximum oxygen consumption, *W*_max_, maximum Watt.

**Table 3:** Influence of exercise on glucose uptake-related signaling pathways in animal models.

Reference	Animals, *n*	Age, week	Training modality	Type of sport	Training frequency	Acute/chronic exercise	Training intensity	Tissue & condition	Time since the last exercise bout, h	Changes in glucose uptake-related molecular signaling^*∗*^
Treadway et al., 1989 [17]	Male Sprague-Dawley rats	n.r.	ET	Treadmill running	45 min	Acute	18 m/min	Insulin stimulated muscle	Immediately after exercise	No effect on insulin binding, basal and insulin-stimulated receptor autophosphorylation, or basal and insulin-stimulated exogenous kinase activity

Goodyear et al., 1995 [18]	Male Sprague-Dawley rats	n.r.	ES	Contraction	n.r.	Acute	Training duration, 500 ms; pulse rate, 100 Hz; duration, 0.1 ms at 1–3 V	Insulin stimulated muscle	Immediately after contraction phase	Decrease of insulin-stimulated tyrosine phosphorylation and PI3-kinase activity (20%), no effect of exercise without insulin

Sakamoto et al., 2002 [50]	Male Sprague-Dawley rats	n.r.	ES	Contraction	n.r.	Acute	Training rate, 1/s; train duration, 500 ms; pulse rate, 100 Hz; duration, 0.1 ms at 2–5 V	Muscle	Immediately after contraction phase	Increase of Akt Ser473 phosphorylation after 5 min (3-fold) and decrease to +23% after 30 min

Wojtaszewski et al., 1999 [51]	Male muscle-specific insulin receptor knockout mice	9-10	ET	Treadmill running	60 min	Acute	22 m/min with 10% incline	Insulin stimulated muscle	Immediately after exercise	Increase of insulin-stimulated glucose transport without improvement of proximal insulin signaling, but increase of Akt phosphorylation (6.0-fold)

Castorena et al., 2014 [49]	Male Wistar rats (LFD and HFD)	n.r.	ET	Swimming	4 × 30 min	Acute	n.r.	Insulin stimulated muscle	Immediately and 3 h after exercise phase	Increase of AS160 immediately (2.0–2.5-fold) and after 3 h (3-fold, in LFD)

Bruss et al., 2005 [57]	Male Wistar rats	n.r.	ES	Contraction	n.r.	Acute	Training rate, 2/min; training duration, 10 s; pulse rate, 100 Hz; duration, 0.1 ms at 2–5 V	Muscle	Immediately after contraction phase	Increase of AS160 phosphorylation (3.7-fold)

Fujii et al., 2005 [97]	Muscle-specific transgenic knockout of *α*2 subunits of AMPK mice	10–16	ES	Contraction	10 min	Acute	Training rate, 1/min; training duration, 10 s; pulse rate, 100 Hz; duration, 0.1 ms at 100 V	Muscle	Immediately after contraction phase	Near normal glucose uptake (−13%) in KO mice

Jeppesen et al., 2013 [98]	Muscle specific knockout of LKB1 mice	16–20	ET	Treadmill running	24 min	Acute	12.5 m/min	Muscle	Immediately after contraction phase	Normal glucose uptake in LKB1 deficient mice

Lefort et al., 2008 [99]	Muscle-specific transgenic knockout of *α*2 subunits of AMPK mice	n.r.	ES	Contraction	2 min	Acute	Training rate, 1/s; training duration, 500 ms; pulse rate, 100 Hz; at 30 V	Muscle	Immediately after contraction phase	No change of AMPK activity after contraction, but increase of glucose uptake by 50% compared to CON mice

Sakamoto et al., 2005 [100]	Muscle specific knockout of LKB1 mice	n.r.	ES	Contraction	5 min	Acute	Training rate, 1/s; training duration, 200 ms; pulse rate, 50 Hz; duration, 0.1 ms at 2–5 V	Muscle	Immediately after contraction phase	Reduced glucose uptake in LKB1 deficient mice

Thomson et al., 2008 [41]	Fischer 344 × Brown Norway male rats	32	ES	Contraction	22 min	Acute	10 sets 6 contractions for 3 s	Muscle	Immediately and 20, and 40 min after contraction phase	Increase of AMPK activity and inhibition of mTOR signaling

Katta et al., 2009 [36]	12 male lean normal Zucker rats, 12 male young obese Syndrome × Zucker rats	10	ES	n.r.	22 min	Acute	10 sets of 6 contractions	Muscle	Immediately, 1 h and 3 h after exercise	Increase of mTOR phosphorylation (Ser2448, 63%) and p70S6K (Thr389, 37%) compared to lean normal Zucker rats

Sylow et al., 2013 [101]	Female C57BL/6 mice	12–16	ET	Treadmill running	50%–70% maximal running speed 30 min	Acute	16 m/min 22 m/min	Muscle	Immediately after exercise	Increase of Rac1 activity by 44%/50%/100% after 40%/50%/70% of maximal speed

Witczak et al., 2007 [102]	Female ICR mice	8	ES	Contraction	15 min	Acute	n.r.	Muscle	45 min after contraction	No change in insulin-stimulated glucose uptake in calmodulin-binding domain-mutant mice, decrease of contraction-stimulated glucose uptake in calmodulin-binding domain-mutant mice

Witczak et al., 2010 [103]	Female ICR mice	6–8	ES	Contraction	10 min	Acute	Training rate, 1/min; training duration, 10 s; pulse rate, 100 pulses/s; duration, 0.1 ms; volts, 100 V	Muscle	45 min after contraction	Decrease of contraction-induced muscle glucose uptake (30%)

Edgett et al., 2013 [46]	Female Sprague-Dawley rats	n.r.	ET	Treadmill running	120 min	Acute	15 m/min + 5 m/min every 5 min	Muscle	Immediately and 3 h after exercise	Time-dependent increase of mTOR mRNA by 44% after 180 min of recovery

Edgett et al., 2013 [46]	Female Sprague-Dawley rats	n.r.	ES	Contraction	120 min	Chronic (7 days)	n.r.	Muscle	Immediately and 3 h after exercise	Increase of mTOR phosphorylation by 74% after 7 days of ES

Calegari et al., 2011 [31]	20 male Wistar rats	8	ET	Treadmill running	5–60 min	Chronic (8 weeks)	5 m/min–30 m/min	Pancreatic islets	24 h after exercise	Increase of AMPK phosphorylation (100%) and CaMKII phosphorylation (+50%)

Luo et al., 2013 [30]	Male Sprague-Dawley rats	18–20	RT	Ladder climbing with weights	3 × 10 repetitions	Chronic (9 weeks)	10% per week increase of additional weight	Muscle	48 h after exercise	Increase of both total and phosphorylated AMPK compared to sedentary control

Ritchie et al., 2014 [53]	Male wild-type (WT, C57BL/6J) mice, adiponectin knockout (AdKO, B6.129-Adipoqtm1Chan/J) mice	12	ET	Treadmill running	3 × 45–60 min	Chronic (8 weeks)	20–32 m/min	Insulin stimulated muscle	48 h after exercise	Increase in total AS160 phosphorylation from AdKO (44%) compared to WT mice (28%); no differences in total GLUT4 protein content

^*∗*^All changes given in the table were statistically significant; AMPK, AMP-activated protein kinase; AS160, Akt substrate of 160 kDa; Ca, calcium; CaMKII, Ca^2+^/calmodulin-dependent protein kinase 2; CON, controls; DIO, diet-induced obesity; ES, electrical stimulation; ET, endurance training; GLUT4, glucose transporter 4; HFD, high fed diet; LFD, low fed diet; LKB-1, liver kinase B1; mTOR, mammalian target of rapamycin (C1 complex 1 & C2 complex 2); n.r., not reported; PI3-K, phosphoinositide 3-kinase; Rac1, ras-related C3 botulinum toxin substrate 1; RT, resistance training; SK6, serine kinase 6; T2D, type 2 diabetes; TBC1D1, TBC1 domain family member 1.

**Table 4:** Influence of exercise on inflammatory signaling and adiponectin in humans.

Reference	Study population, *n*	Age, years	Training modality	Type of sport	Training frequency	Acute/chronic exercise	Training intensity	Tissue & condition	Time since the last exercise bout, h	Changes in cytokines and related inflammatory signaling^*∗*^
Lancaster et al., 2005 [64]	11 trained CON	25 ± 1	ET	Cycling	90 min	Acute	65% VO_2max_ + 34°C radiation	Plasma	Immediately and 2 h after exercise	Increase in IL-6 plasma levels in response to LPS stimulation after exercise

Leggate et al., 2010 [73]	11 trained CON	22 ± 4	ET	Cycling	60 min	Acute	62% VO_2max_ (matched work)	Plasma	Immediately, 1.5, 6 and 23 h after exercise	Increase of soluble interleukin-6 receptor complex after continuous ET (126%)

Leggate et al., 2010 [73]	11 trained CON	22 ± 4	HIT	Cycling	4 min work/2 min rest	Acute	88% VO_2max_ (matched work)	Plasma	Immediately, 1.5, 6 and 23 h after exercise	Increase of soluble interleukin-6 receptor complex plasma levels (159%) and increase of IL-6 plasma levels (2.5-fold) immediately after HIT

Lyngsø et al., 2002 [70]	9 CON	24 ± 1	ET	Cycling	60 min	Acute	60% VO_2max_	Plasma	During, immediately and 3 h after exercise	Increase of IL-6 plasma levels (17-fold) during and 30 min after exercise

Keller et al., 2001 [104]	6 untrained CON	26 ± 4	ET	Two-legged knee extensor apparatus	180 min	Acute	60% of maximum workload of 2 min	Plasma	Immediately, 30, 60, 90 and 180 min after exercise	Increase of IL-6 and TNF-*α* plasma levels immediately and 2 h after exercise

Febbraio et al., 2004 [105]	6 trained CON	24 ± 1	ET	Cycling	120 min	Acute	40% VO_2max_ 70% VO_2max_	Plasma	During (every 30 min), immediately, 60 and 120 min after exercise	Increase of IL-6 plasma levels at 70% of VO_2max_ 60 min after exercise, no change at 40% of VO_2max_

Ostrowski et al., 1998 [72]	16 trained CON	31 ± 2	ET	Marathon	42.2 km	Acute	n.r.	Plasma	Immediately and 2 h after exercise	Increase of IL-6 (62.0-fold), IL-1 receptor antagonist (23.0-fold), TNF-*α* (2.0-fold), and IL-1*β* (1.5-fold) plasma levels immediately after exercise

Ostrowski et al., 1999 [106]	10 trained CON	28 ± 5	ET	Marathon	42.2 km	Acute	n.r.	Plasma	Immediately, and every 30 min until 4 h after exercise	Increase of IL-6 plasma levels (128.0-fold) peaked immediately after exercise and increase of IL-1 receptor antagonist (39.0-fold), TNF-*α* (2.0-fold), and IL-1*β* (2-fold) plasma levels peaked 1 h after exercise

Starkie et al., 2001 [107]	5 trained CON	n.r.	ET	Marathon	150–200 min	Acute	n.r.	Plasma	Immediately, 2 h and 24 h after exercise	Increase of IL-6 and TNF-*α* plasma levels

Oliveira and Gleeson, 2010 [65]	9 trained CON	25 ± 5	ET	Cycling	90 min	Acute	75% VO_2max_	Plasma	Immediately, 2 and 4 h after exercise	Decrease of monocyte TLR4 protein content expression immediately (32%) and 1 h (45%) after exercise

Galpin et al., 2012 [84]	9 trained CON	n.r.	RT	Dynamic pull exercise	15 sets × 3 repetitions	Acute	85% 1-RM	Muscle	During and immediately after exercise	Increase of MAPK (3-fold) and JNK (2.4-fold) phosphorylation

Suzuki et al., 2000 [108]	16 trained CON	n.r.	ET	Marathon	n.r.	Acute	n.r.	Plasma	Immediately after exercise	Increase of IL-6 and IL-1 receptor antagonist plasma levels by 100-fold, decrease of IL-2 by 32% after exercise

Boppart et al., 2000 [83]	14 trained CON	32 ± 2	ET	Marathon	42.2 km	Acute	n.r.	Muscle	Immediately, 1 day, 3 days and 5 days after exercise	Increase of JNK activity immediately after exercise (5-fold), but diminished in the following days

Aronson et al., 1998 [82]	8 CON	30 ± 12	ET	Cycling	60 min	Acute	70% VO_2max_	Muscle	Immediately after exercise	Increase of JNK activity immediately after exercise (6-fold)

Punyadeera et al., 2005 [87]	10 trained CON	23 ± 1	ET	Cycling	120 min	Acute	50% *W*_max_	Plasma & muscle	Immediately and 2 h after exercise	No change in adiponectin plasma levels and adiponectin receptor expression in muscle

Jürimäe et al., 2006 [86]	8 trained CON	63 ± 1	ET	Rowing	6.5 km	Acute	76% VO_2max_	Plasma	Immediately and 30 min after exercise	Increase of adiponectin plasma levels (15%) 30 min after exercise

Fatouros et al., 2005 [93]	50 untrained CON	65–78	RT	Weight machine	3 × 60 min	Chronic (24 weeks)	3-4 sets of 4–12 repetitions with 45–85% of 1-RM	Plasma	48 h after exercise	Increase of adiponectin plasma levels in high-intensity group (60%) and medium-intensity group (18%), still elevated in HI group after 24 weeks of detraining (32%)

Kriketos et al., 2004 [88]	19 sedentary obese CON	37 ± 1	ET	Brisk walking/jogging	4-5 × 40 min	Chronic (10 weeks)	55–70% VO_2max_	Plasma	48 h after exercise	Increase of adiponectin plasma levels by 230%

Lim et al., 2008 [89]	36 CON (young), 38 CON (middle-aged)	22 ± 3 60 ± 6	ET	Cycling	3 × 60 min	Chronic (10 weeks)	70% VO_2max_	Plasma	Immediately after exercise	Increase of adiponectin plasma levels in young (20%) and middle-aged women (27%)

Kondo et al., 2006 [92]	8 untrained obese CON, 8 lean untrained CON	18 ± 1 18 ± 2	ET	Walking/jogging	4-5 × 30 min	Chronic (28 weeks)	60–70% HRR (400–500 kcal)	Plasma	Immediately after exercise	Increase of adiponectin plasma levels in obese CON (75%) and no change in lean CON; decrease of TNF*α* plasma levels in obese CON (37%) and no change in lean CON

Rodriguez-Miguelez et al., 2014 [66]	16 untrained CON	70 ± 1	RT	Leg press, pec deck, biceps curl	2 × 3 sets per 3 exercises 8–12 repetitions	Chronic (8 weeks)	50–80% 1-RM	Plasma	5-6 days after training	Decrease of TLR2 and TLR4 protein content expression and no change in TNF-*α* protein content; upregulation of IL-10 mRNA und protein content after exercise

O'Leary et al., 2006 [90]	16 untrained obese CON	63 ± 1	ET	Running/cycling	5 × 60 min	Chronic (12 weeks)	85% HR_max_	Plasma	18 h after exercise	No change in adiponectin plasma levels

Kadoglou et al., 2007 [78]	30 untrained T2D	57 ± 7	ET	Walking, running, cycling	4 × 45–60 min	Chronic (16 weeks)	50–85% VO_2max_	Plasma	48 h after exercise	Decrease of IL-6 (33%) and IL-18 (40%) plasma levels in T2D after exercise

Leick et al., 2007 [79]	13 untrained obese CON, 16 untrained CON	36 ± 4 25 ± 1	ET	Cycling Rowing	90–120 min 3 × 30 min	Acute Chronic (8 weeks)	60–70% VO_2max_ >70% VO_2max_	Adipose tissue	Immediately, 2 and 10 h after exercise 48 h after exercise	No change of IL-18 mRNA expression after acute exercise in each time point; decrease of IL-18 mRNA (20%) in adipose tissue after exercise

Sriwijitkamol, et al., 2006 [63]	8 untrained CON, 6 untrained T2D	36 ± 3 45 ± 3	ET	Cycling	4 × 45 min	Chronic (8 weeks)	70% VO_2max_	Muscle	24–36 h after exercise	Increase in I*κ*B*α* und I*κ*B*β* protein in CON and T2D (50%) and decrease of TNF*α* protein content in T2D (40%)

Gray et al., 2009 [109]	24 untrained CON	49 ± 9	ET	Community-based walking	5 times	Chronic (12 weeks)	>3000 steps per day	Plasma	n.r.	No change in IL-6, TNF-*α* and hs-CRP plasma levels

Data are given as mean ± SD for age; ^*∗*^all changes given in the table were statistically significant; CON, controls; ET, endurance training; HI, high-intensity; HIT, high-intensity interval training, HR_max_ maximum heart rate, HRR, heart rate reserve; hs-CRP, high-sensitive C-reactive protein; I*κ*B*α*/*β*, nuclear factor of kappa light polypeptide gene enhancer in B-cells inhibitor, alpha/beta; IL-2, interleukin 2; IL-6, interleukin 6; IL-10, interleukin 10; IL-18, interleukin 18; JNK, C-Jun N-terminal kinase; MAPK, mitogen-activated protein kinase; mRNA, messenger RNA; n.r., not reported; RM, one repetition maximum; RT, resistance training; T2D, type 2 diabetes; TLR2, Toll-like receptor 2, TLR4, Toll-like receptor 4, TNF-*α*, tumor necrosis factor alpha; VO_2max_, maximum oxygen consumption; *W*_max_, maximum Watt.

**Table 5:** Influence of exercise on inflammatory signaling and adiponectin in animal models.

Reference	Animals, *n*	Age, week	Training modality	Type of sport	Training frequency	Acute/chronic exercise	Training intensity	Tissue & condition	Time since the last exercise bout, h	Changes in cytokines and related inflammatory signaling^*∗*^
Oliveira et al., 2011 [69]	Male Wistar rats with HFD	8	ET	Swimming	2 × 180 min	Acute	Additional weight of 5% of body weight	Adipose, muscle & hepatic tissue	2, 16, 24, and 36 h after exercise	Decrease in TLR4 mRNA and protein expression in all tissues and reduction in JNK and IKK*β* phosphorylation in adipose, muscle & hepatic tissue; decrease of TNF-*α* and IL-6 mRNA levels in all tissues

Castellani et al., 2015 [74]	Male untrained C57BL/6J mice, trained male C57BL/6J mice	10 14	ET	Treadmill running	120 min	Acute	15 m/min–19 m/min (50% maximal running speed)	Adipose tissue & plasma	Immediately and 4 h after exercise	Increase of IL-6 and IL-6 R*α* protein expression (3-fold) after exercise, more pronounced in trained mice compared to untrained mice

Whitham et al., 2012 [81]	Male untrained C57BL/6 mice (CON), male untrained C57BL/6 mice with JNK-KO (JNK-KO)	n.r.	ET	Treadmill running	30–60 min	Acute	0.22–0.25 m/s	Muscle	Immediately and 30 min after exercise	Increase of muscle IL-6 mRNA expression 30 min after exercise in CON; no change of muscle IL-6 mRNA expression 30 min after exercise in JNK-KO

Macpherson et al., 2015 [75]	Male untrained C57BL/6J mice fed with HFD	7	ET	Treadmill running	120 min	Acute	15 m/min - 5% incline	Adipose tissue	Immediately and 2 h after exercise	Increase of MCP-1 mRNA (2-fold) immediately after exercise and increase of IL-6, MCP-1 (10-fold) and IL-10 (5-fold) mRNA after 2 hours

Kawanishi et al., 2013 [62]	12 male C57BL/6J mice with HFD, 12 C57BL/6J mice with ND	4	ET	Treadmill running	5 × 60 min	Chronic (16 week)	15 m/min–20 m/min	Adipose tissue & liver	72 h after exercise	Higher levels of TNF*α* mRNA (4.0-fold) and IL-6 mRNA (2.5-fold) in HFD sedentary mice compared to ND mice after chronic exercise

Cho et al., 2016 [94]	10 untrained C57BL/6 mice with HFD	15	HIT	Treadmill running	40 min	Chronic (8 weeks)	10–17 m/min	Muscle	Immediately after exercise	Prevention of downregulation of AdipoR1 expression caused by HFD

Ritchie et al., 2014 [53]	Male wild-type (WT, C57BL/6J), adiponectin knockout (AdKO, B6.129-Adipoqtm1Chan/J) mice	12	ET	Treadmill running	3 × 45–60 min	Chronic (8 weeks)	5 × 20–32 m/min	Muscle	48 h after exercise	Increase in total AS160 from AdKO (44%) compared to WT mice (28%); no differences in total GLUT4

da Luz et al., 2011 [67]	Obese DIO rats	n.r.	ET	Swimming	5 × 60 min	Chronic (8 weeks)	Additional weight of 5% of body weight	Adipose tissue & hepatic tissue	Immediately after exercise	Decrease of JNK, I*κ*B, and NF-*κ*B activity and protein expression and increase of IRS-1, insulin receptor, and Akt phosphorylation after chronic exercise in adipose and hepatic tissue

Medeiros et al., 2011 [68]	Obese Wistar rats with HFD	n.r.	ET	Swimming	n.r.	Chronic (12 weeks)	n.r.	Adipose tissue	n.r.	Increase in Akt (2.3-fold) and Foxo1 (1.7-fold) phosphorylation, reduction in phospho-JNK (1.9-fold), NF-kB (1.6-fold) and PTP-1B (1.5-fold) protein expression, and increase in mTOR (1.7-fold), p70S6k (1.9-fold), and 4E-BP1 phosphorylation (1.4-fold) after exercise training

Oliveira et al., 2011 [69]	Male Wistar rats with HFD	8	ET	Swimming	5 × 60 min	Chronic (8 weeks)	Additional weight of 5% of body weight	Adipose, muscle & hepatic tissue	24 and 36 h after exercise	Decrease in TLR4 mRNA and protein expression and reduction of JNK and IKK*β* phosphorylation in adipose, muscle & hepatic tissue; Increase of insulin-stimulated IRS-1 and insulin receptor and Akt phosphorylation, decrease of TNF*α* and IL-6 mRNA level in all tissues

Passos et al., 2015 [85]	Male Sprague-Dawley rats with HFD	5-6	ET	Treadmill running	5 × 60 min	Chronic (8 weeks)	15–25 m/min	Plasma	Immediately after exercise	Decrease in JNK activation and total JNK level in HFD compared to sedentary HFD

Mardare et al., 2016 [80]	Male C57BL/6 mice	10	ET	Treadmill running	5 × 30 min	Chronic (10 weeks)	80% VO_2max_	Serum & adipose tissue	72 h after exercise	Decrease of IL-18 and TNF-*α* expression in adipose tissue

Mardare et al., 2016 [80]	Male C57BL/6 mice	10	RT	Isometric strength training	5 × 3 min with 3 sets	Chronic (10 weeks)	n.r.	Serum & adipose tissue	72 h after exercise	Decrease of IL-18 serum levels

^*∗*^All changes given in the table were statistically significant; CON, controls; DIO, diet-induced obesity; ES, electrical stimulation; ET, endurance training; GLUT4, glucose transporter 4; HFD, high fed diet; HI, high-intensity; HIT, high-intensity interval training; hs-CRP, high-sensitive C-reactive protein; I*κ*B*α*/*β*, nuclear factor of kappa light polypeptide gene enhancer in B-cells inhibitor, alpha/beta; IL-6, interleukin 6; IL-6 R*α*, interleukin 6 receptor *α*; IL-10, interleukin 10; IRS-1, insulin receptor substrate 1; JNK, C-Jun N-terminal kinase; MCP-1, monocyte chemotactic protein 1; mRNA, messenger RNA; ND, normal diet; NF-*κ*B, nuclear factor kappa; n.r., not reported; RT, resistance training; TNF-*α*, tumor necrosis factor alpha; TLR4, Toll-like receptor 4.
